# Multinational cost-effectiveness analysis of pembrolizumab combined with chemotherapy as first-line treatment for advanced biliary tract cancer

**DOI:** 10.3389/fpubh.2025.1597550

**Published:** 2025-08-11

**Authors:** Yuxuan Xie, Quanyi Liu, Shanrui Xiao, Xia Li, Lin Qiu, Yun Gu

**Affiliations:** ^1^Department of Pharmacy, The First Affiliated Hospital of Dali University, Dali, China; ^2^College of Pharmacy, Dali University, Dali, China

**Keywords:** biliary tract cancer, pembrolizumab, immune checkpoint inhibitors, multinational economic evaluation, cost-effectiveness analysis

## Abstract

**Background:**

Immunotherapy is a promising treatment for advanced biliary tract cancer. However, the cost-effectiveness of pembrolizumab combined with gemcitabine and cisplatin remains unclear across different healthcare systems. This study evaluates its international cost-effectiveness in four countries.

**Methods:**

A partitioned survival analysis model was developed using data from the KEYNOTE-966 trial to compare costs, quality-adjusted life years (QALYs), and incremental cost-effectiveness ratio (ICER) of pembrolizumab plus gemcitabine and cisplatin versus gemcitabine and cisplatin alone, from the healthcare system perspective of China, Japan, the United States, and Switzerland. Sensitivity and scenario analyses were used to identify key factors influencing the ICER.

**Results:**

The base-case analysis showed that pembrolizumab plus gemcitabine and cisplatin provided additional quality-adjusted life year gains of 0.14 in China, Japan, and the United States, and 0.15 in Switzerland. The incremental cost-effectiveness ratios in all four countries exceeded their respective willingness-to-pay thresholds, indicating limited cost-effectiveness. Sensitivity analysis identified drug price and utility value of progression-free survival as key factors. In the United States, Japan, and Switzerland, an 80–95% reduction in pembrolizumab’s price was necessary for cost-effectiveness, while in China, a reduction greater than 95% was required.

**Conclusion:**

Despite clinical benefits, pembrolizumab combined with chemotherapy for advanced biliary tract cancer is not cost-effective at current prices and willingness-to-pay thresholds. Adjusting drug pricing and healthcare policies is crucial for enhancing the global economic viability of this treatment strategy.

## 1 Introduction

Biliary tract cancer (BTC) encompasses intrahepatic cholangiocarcinoma, perihilar and distal cholangiocarcinoma, and gallbladder cancer, characterized by its aggressive nature and poor prognosis (1). Due to the obvious heterogeneity of BTC and its subtle clinical symptoms (2), more than 70% of patients are diagnosed at an advanced stage, which limits the available treatment options. The five-year survival rate is below 5%, presenting a considerable challenge for treatment (3–5).

The incidence of BTC varies greatly worldwide. Countries in East Asia, including China, Thailand, and South Korea, exhibit incidence rates significantly exceeding the global average, approximately 40 times higher than those observed in high-income nations (6). This creates considerable health burdens and results in significant socioeconomic and healthcare system challenges for the country.

Currently, the standard treatment for advanced biliary tract cancer (ABTC) is chemotherapy, specifically gemcitabine combined with cisplatin (GP) (7). Although GP has demonstrated an ability to prolong survival, its efficacy is limited, and it often causes severe side effects (8), such as myelosuppression, gastrointestinal complications, and immune suppression, which adversely impact patients’ quality of life. Consequently, there is a pressing requirement for more efficient treatments with fewer side effects.

Immune checkpoint inhibitors (ICIs) have recently represented a significant advancement in cancer therapy. PD-1 inhibitors (Programmed Cell Death Protein 1 Inhibitor) enhance T-cell-mediated anti-tumor immune responses by blocking the PD-1/PD-L1 pathway (9). Pembrolizumab, a PD-1 inhibitor, has demonstrated notable efficacy across various cancers, including non-small cell lung cancer, esophageal cancer, and head and neck cancer (10–12). The KEYNOTE-966 trial demonstrated that pembrolizumab plus GP could be an effective first-line treatment for patients with ABTC. It resulted in a median overall survival (OS) of 12.7 months and a median progression-free survival (PFS) of 6.5 months, along with improvements in quality of life (QoL) (13). Several national treatment guidelines, such as the 2025 Chinese Society of Clinical Oncology (CSCO) Guidelines and the 2025 National Comprehensive Cancer Network (NCCN) Guidelines (Version 3.0), have included this treatment as a first-line therapy for ABTC. Despite its clinical efficacy, pembrolizumab is a costly therapy characterized by prolonged treatment durations and moderate survival benefits. The uncertainty surrounding its cost-effectiveness restricts its broader application.

Variations in healthcare systems, medical expenses, and payment mechanisms among countries may significantly influence the cost-effectiveness of immune checkpoint inhibitors. Currently, there is limited global economic evaluation of ABTC treatment, as existing cost-effectiveness studies primarily focus on individual countries or a select few, leading to inconsistent conclusions. This study employs a partitioned survival analysis model (PartSA) to conduct a cross-country cost-effectiveness evaluation, incorporating treatment cost and utility data from China, Japan, the United States, and Switzerland. These countries represent varying levels of economic development and healthcare systems. Within this comparative framework, the study aims to assess the economic feasibility and applicability of pembrolizumab in combination with GP for the treatment of ABTC, thereby providing valuable insights to inform clinical decision-making, drug pricing, and policy development.

## 2 Method

### 2.1 Target population and clinical data

This study conducted a cost-effectiveness analysis utilizing data from the KEYNOTE-966 trial, a multicenter, randomized, double-blind, placebo-controlled phase III clinical trial. Although Switzerland was not a participant in the KEYNOTE-966 trial, pembrolizumab has been approved for use in the country. Switzerland was included in our cost-effectiveness analysis as a representative high-income setting with transparent pharmaceutical pricing and well-documented health economic parameters. We aligned the target population with the trial cohort, which included patients with previously untreated, unresectable, locally advanced, or metastatic extrahepatic or intrahepatic biliary tract cancer (including gallbladder cancer), exhibiting measurable lesions as defined by the Response Evaluation Criteria in Solid Tumors (RECIST). All patients enrolled had an Eastern Cooperative Oncology Group (ECOG) performance status of one or two, maintained normal organ function, and had an anticipated survival exceeding 3 months.

The KEYNOTE-966 trial comprised 1,069 patients who were randomized into two groups: 533 patients received pembrolizumab (200 mg), while 536 patients received a placebo (200 mg). Each treatment cycle spanned 21 days, with a maximum of 35 cycles for either pembrolizumab or placebo. Both groups also received intravenous gemcitabine (1,000 mg/m^2^) and cisplatin (25 mg/m^2^) on Days 1 and 8 of each cycle, with cisplatin limited to a maximum of 8 cycles. Patients with progressive disease (PD) or intolerable toxicity were transitioned to second-line or alternative therapies.

We chose the FOLFOX regimen (oxaliplatin, leucovorin calcium, and fluorouracil) as the second-line therapy, in line with treatment guidelines from the four countries we analyzed. According to the KEYNOTE-966 data, 47% of patients in the pembrolizumab group and 49% in the placebo group underwent second-line treatments. Furthermore, 0.9% of patients in the pembrolizumab group and 1.1% in the placebo group received immunotherapy following disease progression, while the other patients were provided with the best supportive care.

### 2.2 Model structure

A partitioned survival analysis model was created with TreeAge Pro software. The model categorized patients into three health states: PFS, PD, and death. All patients initially presented in the PFS state and had the potential to transition to either the PD or death state over time. Following disease progression, patients in the PD state were presumed to undergo second-line therapies or alternative treatments until death.

**Figure 1:**
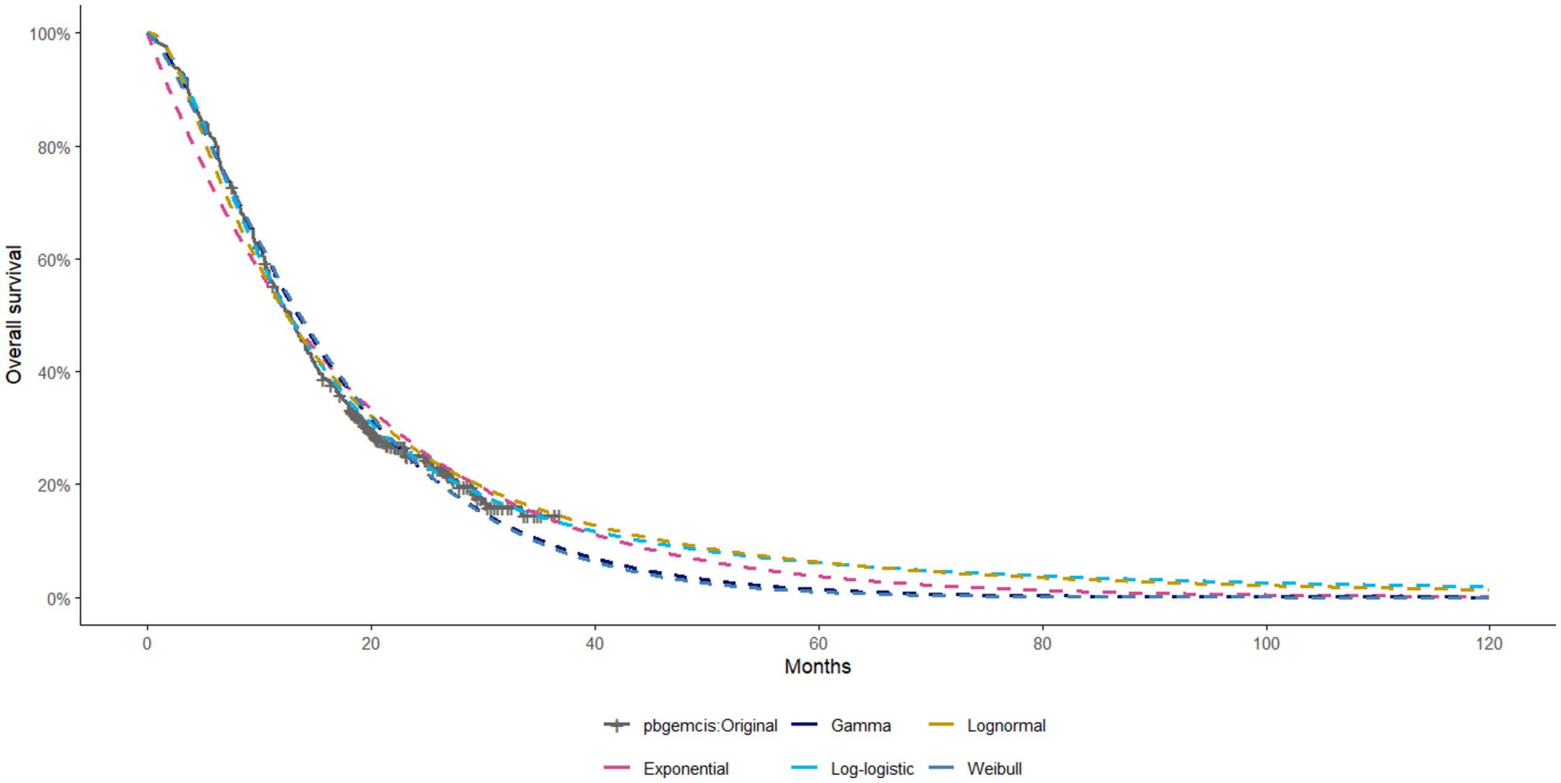
Extrapolated OS Curves of Different Statistical Distributions for Pembrolizumab + Gemcitabine + Cisplatin (Other curves are provided in the Supplementary material).

The model employed a 21-day cycle and a time horizon of 10 years, by which point nearly all patients in both groups had transitioned to the death state. Survival data during the trial follow-up period were directly reconstructed from the Kaplan–Meier curves reported in the KEYNOTE-966 trial. The reconstructed OS and PFS curves are shown in Supplementary Figure S1. For extrapolation beyond the trial period, survival curves were fitted and projected using standard parametric methods. Extrapolated OS curves under different statistical distributions are shown in Figure 1, while additional curves are provided in the Supplementary Figure S2. The assessment of cost-effectiveness involved calculating total costs, quality-adjusted life years (QALYs), and incremental cost-effectiveness ratios (ICERs) for the two treatment strategies.

The willingness-to-pay (WTP) thresholds per QALY were set at $37,663 for China [equivalent to three times China’s per capita GDP in 2024 (14)], $229,044 for the United States [three times the U. S. per capita GDP in 2022 (14)], $49,261 for Japan (15), and $112,250 for Switzerland (16). Exchange rates used were: $1 = 7.1179 CNY; $1 = 0.891 GBP; $1 = 152.25 JPY.

### 2.3 Clinical data input

Survival data for this study were extracted from the KEYNOTE-966 trial utilizing GetData Graph Digitizer software. Individual patient data (IPD) reconstruction was conducted using R version 4.3.3 software. Five parametric survival distributions—Weibull, Gamma, log-logistic, lognormal, and exponential—were fitted to the reconstructed data. The optimal distribution was determined using the Akaike Information Criterion (AIC) and Bayesian Information Criterion (BIC). Supplementary Tables S1–S3 summarize the final selected distributions and their associated parameters.

### 2.4 Costs and utility values

This study focused on direct medical costs incurred during treatment, including drug costs, examination costs, disease management costs, best supportive care costs, adverse event (AE) management costs, and end-of-life care costs. After disease progression, patients could receive FOLFOX as second-line therapy, immunotherapy, or best supportive care. The analysis focused on adverse events classified as grade 3 or higher, with an incidence rate of 9% or greater. These events included thrombocytopenia, leukopenia, neutropenia, and anemia. AE costs were only included for the first treatment cycle.

Drug costs were sourced from various country-specific references: in China, they were based on the 2024 average drug prices listed on Yaozhi.com; in Japan, they were derived from the 2024 drug price list published by the Ministry of Health, Labour and Welfare (MHLW); in the United States, costs were based on the 2022 average spending per dose reported by the Centers for Medicare & Medicaid Services (CMS); and in Switzerland, they were obtained from the most recent data published by the Swiss Federal Office of Public Health.

Furthermore, costs associated with disease management, examination, AE management, best supportive care, and end-of-life care, as well as utility values for different health states and disutility values for AEs, were obtained from published literature. Drug costs were calculated based on the average patient body surface area for each country. Discount rates for costs and utilities were established at 5% for China (17), 3% for Japan (18), 3% for the United States (19), and 3% for Switzerland (20, 21). The model costs were converted to US dollars. Key model parameter values are summarized in Table 1, with corresponding parameter ranges provided in Supplementary Table S4.

**Table 1:** Key model inputs.

Treatment cost	China	Ref.	Japan	Ref.	US	Ref.	CH	Ref.	Distribution
Pembrolizumab (100 mg)	2,517.32	(34)	1,408.85	(35)	5,342.15	(36)	5,399.28	(37)	Gamma
Gemcitabine (0.2 g)	8.43	(34)	6.11	(35)	4.00	(36)	41.36	(37)	Gamma
Cisplatin (10 mg)	2.42	(34)	22.09(50 mg)	(35)	3.20	(36)	35.53	(37)	Gamma
Fluorouracil (250 mg)	8.35	(34)	1.58	(35)	2.10	(36)	15.55 (500 mg)	(37)	Gamma
Leucovorin calcium (0.1 g)	3.66	(34)	2.99	(35)	8.31	(36)	45.01	(37)	Gamma
Oxaliplatin (50 mg)	53.84	(34)	18.10	(35)	6.00	(36)	112.03	(37)	Gamma
Adverse event cost, $									
Neutropenia	354.00	(38)	244.62	(39)	5,321.00	(40)	6,845.62	(41)	Gamma
Leukopenia	466.00	(38)	163.03	(42)	5,321.00	(40)	9,307.84	(43)	Gamma
Thrombocytopenia	1,814.00	(44)	803.63	(21)	6,325.00	(40)	6,845.62	(41)	Gamma
Anemia	541.00	(38)	15.00	(39)	4,953.00	(40)	6,845.62	(41)	Gamma
Other cost, ($ per cycle)									
Examination	55.60	(45)	115.67	(46)	851.00	(47)	659.79	(20, 48)	Gamma
Administration	110.20	(49)	620.00	(50)	324.00	(51)	1,198.83	(41)	Gamma
Best supportive care	337.50	(45)	79.85	(52)	523.48	(51)	249.82	(16)	Gamma
End-of-life care	2,299.00	(53)	12,628.56	(54)	7,894.00	(55)	14,101.41	(16)	Gamma
Utility values									
PFS	0.76	(17)	0.73	(56)	0.76	(55)	0.80	(16)	Beta
PD	0.68	(17)	0.69	(56)	0.68	(55)	0.73	(16)	Beta
BSA (m^2^)	1.72	(57)	1.73	(39)	1.86	(58)	1.93	(20)	
Discount rate	0.05	(17)	0.03	(18)	0.03	(19)	0.03	(20)	
Probability of AEs in pem group									
Neutropenia	0.47							(13)	Beta
Leukopenia	0.12							(13)	Beta
Thrombocytopenia	0.16							(13)	Beta
Anemia	0.24							(13)	Beta
Probability of AEs in the chem group									
Neutropenia	0.46							(13)	Beta
Leukopenia	0.09							(13)	Beta
Thrombocytopenia	0.18							(13)	Beta
Anemia	0.25							(13)	Beta

### 2.5 Sensitivity analysis

Deterministic sensitivity analysis (DSA) and probabilistic sensitivity analysis (PSA) were conducted to evaluate the robustness of the model results. Parameter ranges were determined based on 95% confidence intervals or by varying base-case values by ±20% (see Supplementary Table S4 for further details). Results of the one-way DSA were illustrated by tornado diagrams. Five thousand Monte Carlo simulations were performed for PSA. Gamma distributions were applied to cost parameters, while beta distributions were used for probabilities and utility values. PSA results were illustrated using cost-effectiveness acceptability curves (CEACs) and incremental cost-effectiveness scatterplots (ICE scatterplots).

### 2.6 Scenario analysis

Scenario analysis was conducted to evaluate the effects of pembrolizumab price decreases on cost-effectiveness. Price reductions of pembrolizumab were simulated for China, Japan, the United States, and Switzerland at 10, 20, 30, 40, 50, 60, 70, 80, 90, and 95%. In each price reduction scenario, the ICER was recalculated and compared against the WTP thresholds to assess how price changes affected the economic conclusions.

## 3 Results

### 3.1 Base case analysis

Compared to chemotherapy alone, the additional costs for combination therapy in China, Japan, the United States, and Switzerland were $74,621.78, $44,014.83, $160,169.86, and $163,631.09, respectively; the QALY gains were 0.14, 0.14, 0.14, and 0.15, respectively. The cost per additional QALY gained was $542,441.16, $314,213.67, $1,104,628.40, and $1,069,496.82, respectively. When comparing the ICER values of the four countries with their respective WTP thresholds, the results indicated that the ICER values for combination therapy were significantly higher than the WTP thresholds in all four countries (see Table 2).

**Table 2:** Results of base-case analysis.

Country	Drug	Cost	Incremental cost	Effectiveness	Incremental QALY	ICER^c^
China	pbgemcis^a^	91,983.16	74,621.78	0.98	0.14	542,441.16/QALY
	gemcis^b^	17,361.38		0.84		
Japan	pbgemcis	78,916.38	44,014.83	1.00	0.14	314,213.67/QALY
	gemcis	34,901.55		0.86		
United States	pbgemcis	217,340.46	160,169.86	1.02	0.14	1,104,628.40/QALY
	gemcis	57,170.60		0.88		
Switzerland	pbgemcis	248,359.72	163,631.09	1.09	0.15	1,069,496.82/QALY
	gemcis	84,728.63		0.94		

a pbgemcis, pembrolizumab + gemcitabine + cisplatin.

b gemcis, gemcitabine + cisplatin.

c ICER, incremental cost-effectiveness ratio.

### 3.2 Sensitivity analysis

**Figure 2:**
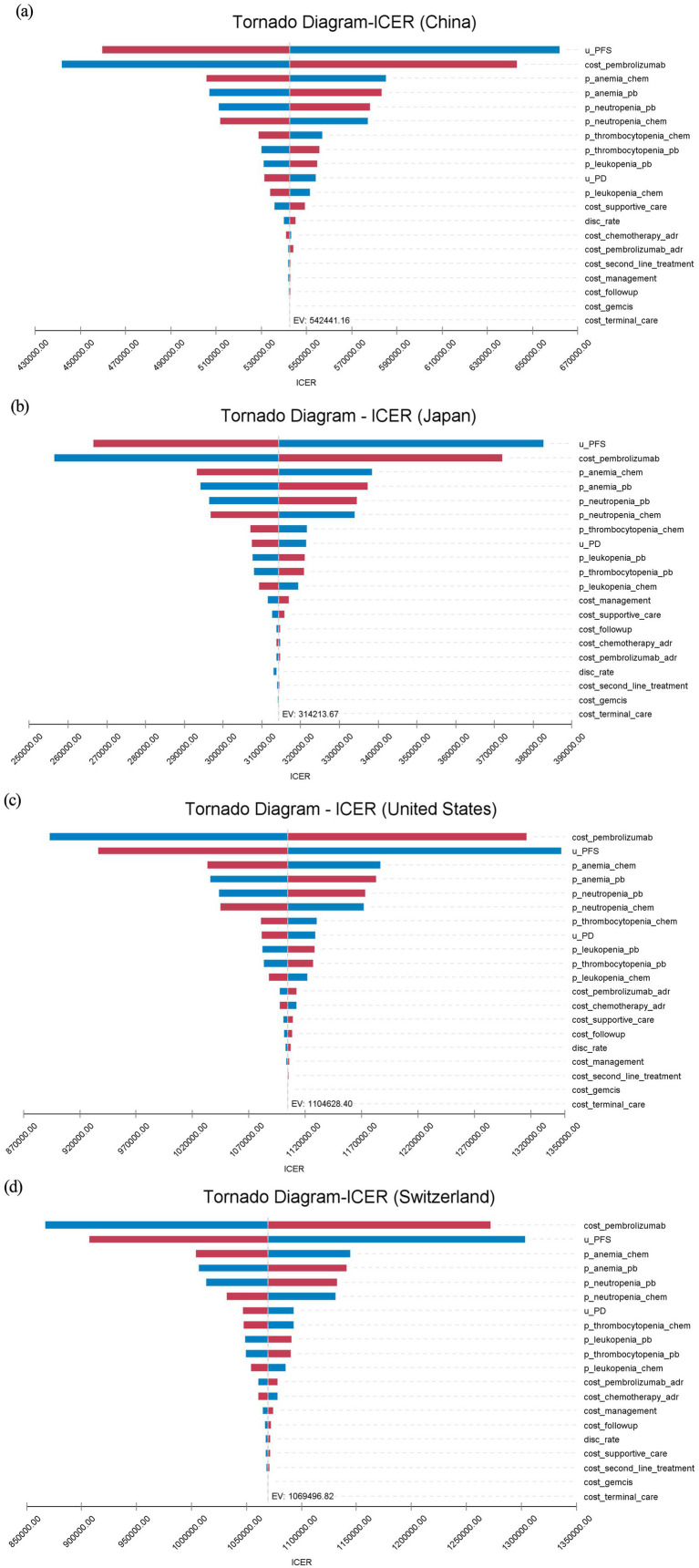
Tornado diagram of one-way deterministic sensitivity analysis.

One-way DSA showed consistent results across countries. The price of pembrolizumab and the utility value for PFS were recognized as critical parameters influencing the ICER. In the United States and Switzerland, the cost of pembrolizumab had the most significant impact on the ICER, while in China and Japan, the PFS utility value had a greater influence on the ICER. Anemia and neutropenia, among other adverse events, also had a significant impact on the ICER results. Within the range of parameter fluctuations, the ICER remained above the WTP thresholds in all countries, consistent with the base case analysis (see Figure 2).

**Figure 3:**
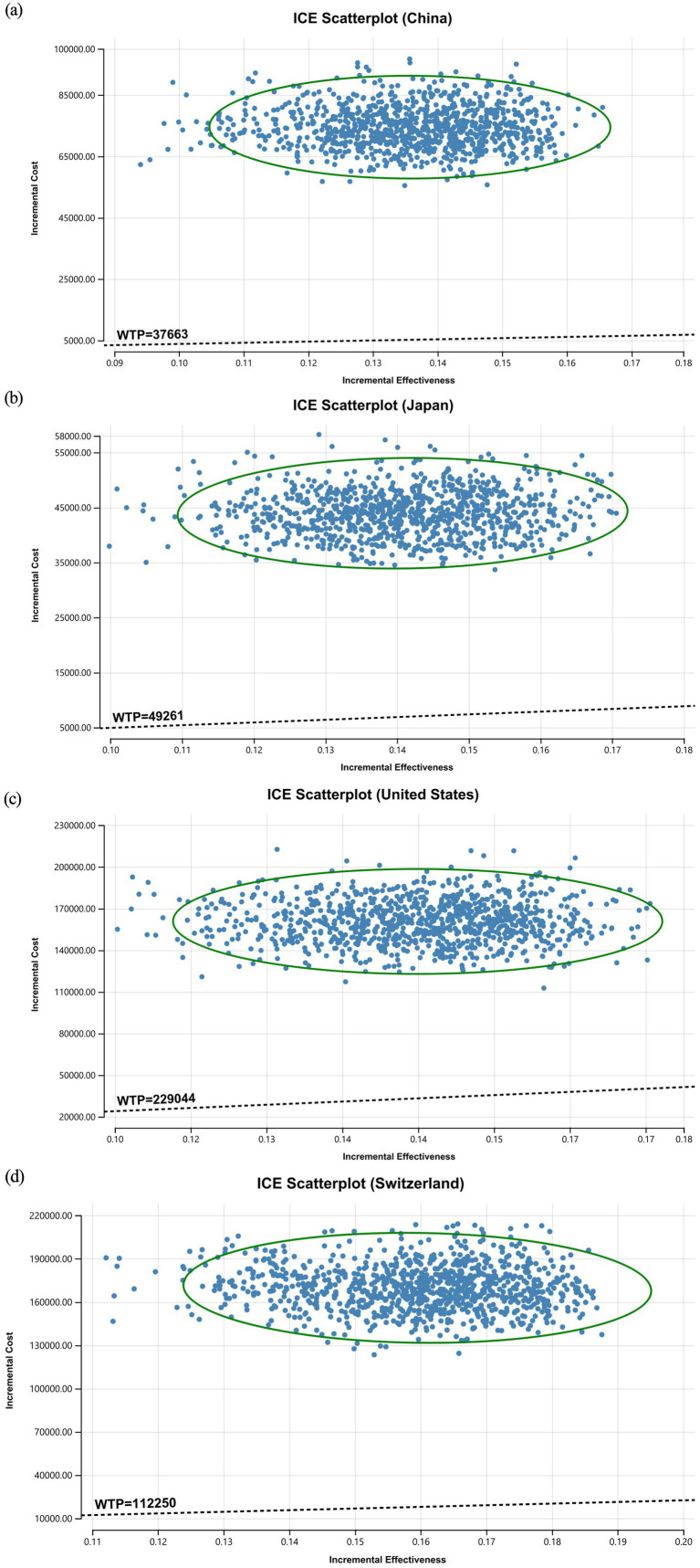
Probability sensitivity analysis (ICE scatterplots).

The ICE scatterplot of the probabilistic sensitivity analysis results showed that all of the data points were in the first quadrant. This indicates that pembrolizumab not only increased QALYs but also increased the cost for all countries. At current WTP thresholds, all data points were above the threshold line (see Figure 3).

The analysis of the cost-effectiveness acceptability curve revealed that the probability of cost-effectiveness for pembrolizumab in combination with chemotherapy was zero at lower WTP levels. As WTP thresholds increased, the chance of cost-effectiveness progressively improved. Only when WTP significantly increased above USD 1,000,000/QALY did we observe cost-effectiveness in Switzerland and the United States. In China and Japan, the WTP turning points were comparatively lower, at approximately USD 550,000/QALY and USD 320,000/QALY, respectively, indicating some degree of cost-effectiveness. Under the existing WTP levels in each country, the likelihood of cost-effectiveness was nonexistent. These results confirm the robustness of the base case analysis (see Supplementary Figure S3).

### 3.3 Scenario analysis

**Figure 4:**
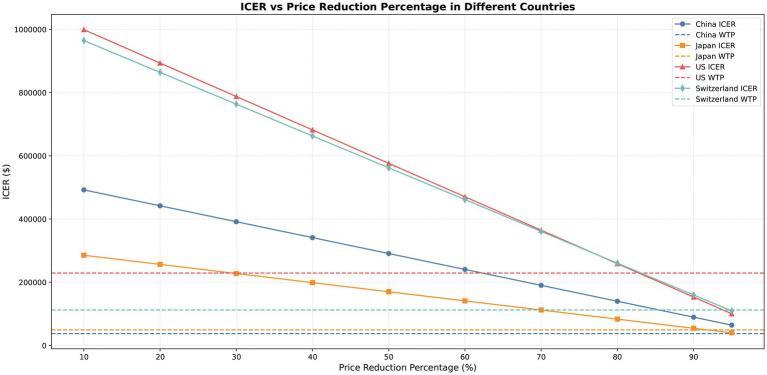
ICER variation under different price reduction scenarios across countries.

The price of pembrolizumab significantly influenced the ICER, with price reductions leading to a gradual decrease. At the WTP thresholds, an 80–90% price reduction in the United States resulted in cost-effectiveness. In Switzerland and Japan, a price reduction of nearly 95% demonstrated economic benefit, while in China, prices needed to decrease by more than 95% for the ICER to approach the WTP threshold (see Figure 4 and Supplementary Table S5).

## 4 Discussion

This study evaluated the cost-effectiveness of pembrolizumab combined with chemotherapy for treating ABTC in China, Japan, the United States and Switzerland. The results showed incremental QALY gains of 0.14 for China, Japan, and the United States, and 0.15 for Switzerland compared to chemotherapy alone. The incremental cost-effectiveness ratios (ICERs) for the four countries were as follows: $542,441.16 for China, $314,213.67 for Japan, $1,104,628.40 for the United States, and $1,069,496.82 for Switzerland, all exceeding their respective WTP thresholds. The results indicate that the treatment is generally not cost-effective under the current economic context.

While our findings are consistent with several prior cost-effectiveness evaluations conducted in China and the United States (22, 23), they differ from the study by Zhu et al. (24), which reported pembrolizumab plus chemotherapy to be cost-effective in China. Although both studies were based on clinical efficacy data from the KEYNOTE-966 trial, the discrepancy in conclusions may be attributed to differences in model structure and cost input sources. Zhu et al. employed a Markov model and derived cost parameters from real-world drug prices in China. In contrast, our analysis applied a partitioned survival model (PSM), used national average drug prices from Yaozhi.com for China, and incorporated officially published pricing data for Japan, the United States, and Switzerland. By implementing a consistent modeling strategy and harmonized cost assumptions across all four countries, this study minimized the impact of structural variability and provides complementary cross-national evidence on the economic value of pembrolizumab. Although the same modeling framework was used across all settings, the observed differences in cost-effectiveness primarily reflect variations in drug pricing, healthcare expenditure levels, and country-specific WTP thresholds.

As the KEYNOTE-966 trial did not report specific utility values and substantial variation exists across national healthcare systems, we adopted utility values from country-specific pharmacoeconomic literature. These values were primarily derived using the EQ-5D instrument and extracted from published economic evaluations of other cancer types. In the absence of BTC-specific utility data, we referenced malignancies such as hepatocellular carcinoma, gastric cancer, and non-small-cell lung cancer, which exhibit partially comparable characteristics in terms of disease progression, treatment strategies, and health-related quality of life (HRQoL) impacts. The slightly higher incremental QALY observed in Switzerland may be attributed to the use of utility values derived from lung cancer, which tend to be higher than those from more comparable cancers. This proxy-based approach has been widely applied in previous pharmacoeconomic studies and is generally considered both methodologically valid and practically feasible (25–27). As a core input in cost-utility analysis, utility values significantly affect model outcomes, as further confirmed by our sensitivity analyses.

The sensitivity analysis indicated that the results were stable, with drug price and the utility value of PFS as the primary drivers of ICER. Future research on BTC-specific utility values may play a key role in improving the accuracy and applicability of related economic evaluations. Scenario analysis simulated price reductions for pembrolizumab ranging from 10 to 95%. The results indicated that a price decrease of 80–95% would be required for the ICER to approach the WTP thresholds in the United States, Japan, and Switzerland, suggesting potential cost-effectiveness under these scenarios. In contrast, for China, price reductions surpassing 95% would be necessary to bring the ICER closer to the WTP thresholds.

In China, the National Healthcare Security Administration (NHSA) negotiates with pharmaceutical companies to include innovative and cost-effective drugs in the National Reimbursement Drug List (NRDL), often resulting in significant price reductions (28). In the 2024 NRDL negotiations, non-reimbursed drugs achieved an average price reduction of 63.0%, and in 2020, the price of camrelizumab dropped by 85.2% after inclusion in the NRDL. Additionally, China’s “1+3+N” model has established a multi-layered healthcare system to ensure universal health coverage. The healthcare system in Japan is distinct from other countries. The Ministry of Health, Labour and Welfare (MHLW) regularly sets and updates drug prices, while national health insurance covers 70–90% of medical costs (29, 30). In the United States, Medicare typically covers 80% of medical expenses, with patients responsible for the remaining 20%. In Switzerland, healthcare reimbursement covers 70–90% of costs exceeding the deductible, with a patient out-of-pocket limit. These strategies help reduce patient financial burdens and improve drug accessibility. However, BTC indications have not yet widely adopted pembrolizumab and other innovative drugs, and reimbursement policies remain insufficient.

The findings of the TOPAZ-1 trial further support the efficacy of immunotherapy in BTC. For instance, durvalumab combined with chemotherapy showed favorable clinical outcomes in ABTC. However, a study by Ye et al. (31) showed that durvalumab combined with chemotherapy was not cost-effective either in the United States or China. Moreover, an international cost-effectiveness study of durvalumab for unresectable non-small-cell lung cancer reported high treatment costs in countries including the United States, Brazil, Singapore, and Spain (32). These findings highlight the misalignment between the high prices of immunotherapies and patients’ financial capacities, which remains a critical barrier to the widespread adoption of innovative treatments. A policy report by the NCCN also pointed out that the combination of high drug prices and delayed reimbursement systems remains a key barrier for cancer patients in accessing innovative treatments, emphasizing the urgent need for targeted solutions (33).

To improve the economic feasibility of immunotherapy regimens, countries should tailor multi-layered policy strategies to their specific healthcare systems. Key measures include strengthening drug price negotiations, adopting outcome-based payment models, expanding reimbursement coverage, and increasing reimbursement rates. Additionally, international collaboration on drug pricing could provide valuable insights, enhancing the accessibility of immunotherapy and reducing the financial burden on patients. These efforts could accelerate the global adoption of immunotherapy for cancer treatment.

This study has several limitations. First, we did not fully account for individual patient differences, disease progression heterogeneity, and BTC subtype diversity. Second, cost and utility data were derived from published literature, which may reflect regional variations. Although using EQ-5D data collected in the KEYNOTE-966 trial would have provided more representative and internally valid utility estimates for each health state, individual-level utility data were not publicly available at the time of our analysis, and EQ-5D-related quality-of-life outcomes had not been published in any Supplementary material or separate articles. Third, adverse event costs were only included for the initial treatment cycle. Differences in exchange rates and healthcare standards across countries may also have influenced the results. In addition, the model did not incorporate real-world long-term follow-up data. Survival curves were constructed based on the overall trial population rather than country-specific subgroups; given the known regional disparities in BTC epidemiology, this may limit the precision of cross-country comparisons.

Future research should include large-scale, multicenter, prospective trials and stratified analyses of BTC patients to provide more comprehensive global cost-effectiveness evaluations. Incorporating country-specific survival data in future models could further enhance the accuracy and comparability of cross-national economic evaluations. Furthermore, integrating trial-based EQ-5D utility data from KEYNOTE-966, once available, will be essential to improve the methodological robustness of health utility estimation.

## 5 Conclusion

Combining pembrolizumab with chemotherapy demonstrates promising clinical efficacy in treating ABTC. Nevertheless, given current drug pricing and WTP thresholds, its cost-effectiveness remains limited. Optimizing drug prices and healthcare policies is essential to enhance the global economic viability of this treatment.

## Data Availability

The original contributions presented in the study are included in the article/Supplementary material, further inquiries can be directed to the corresponding author.
