# Supplementary material for: Multinational cost-effectiveness analysis of pembrolizumab combined with chemotherapy as first-line treatment for advanced biliary tract cancer
Source: Front Public Health. 2025 Aug 11;13:1597550. doi: 10.3389/fpubh.2025.1597550 (PMC12375827; doi:10.3389/fpubh.2025.1597550)
Supplement: Supplementary file 1 [file Data_Sheet_1.docx]

Multinational cost-effectiveness analysis of pembrolizumab combined with chemotherapy as first-line treatment for advanced biliary tract cancer**----*Supplementary Materials***

**Table S1.** Survival inputs: Kaplan-Meier curve fitting parameters

**Table S2.** AIC and BIC values of OS curve fitting distributions

**Table S3.** AIC and BIC values of PFS curve fitting distributions

**Table S4.** The range of key model inputs

**Table S5.** Scenario analysis

**Figure S1.** OS and PFS Curves Reconstructed from the KEYNOTE-966 Phase III Trial

**Figure S2.** Extrapolation Curves of Different Statistical Distributions

**Figure S3.** Probability sensitivity analysis (CEACs)

**Table S1** Survival inputs: Kaplan-Meier curve fitting parameters

| **Treatment Regimen Curve** | **AIC** | **BIC** | **Best Fit Distribution** | **Parameters** |
| --- | --- | --- | --- | --- |
| Pbgemcis PFS | 2291.845 | 2300.402 | Lognorml | meanlog = 1.788; sdlog = 1.121 |
| Pbgemcis OS | 3161.904 | 3170.461 | Loglogistic | shape = 1.758; scale = 12.668 |
| gemcis PFS | 2316.106 | 2324.674 | Lognorml | meanlog = 1.662; sdlog =1.003 |
| gemcis OS | 3246.259 | 3254.828 | Loglogistic | shape = 1.761; scale = 10.896 |

***** Pbgemcis, Pembrolizumab + Gemcitabine + Cisplatin

gemcis, Gemcitabine + Cisplatin

**Table S2** AIC and BIC values of OS curve fitting distributions for the two treatment groups

| **Model** | **Pbgemcis, AIC** | **Pbgemcis, BIC** | **Gemcis, AIC** | **Gemcis, BIC** |
| --- | --- | --- | --- | --- |
| exponential | 3209.897 | 3214.176 | 3287.086 | 3291.371 |
| Weibull | 3178.650 | 3187.207 | 3256.349 | 3264.917 |
| Gamma | 3171.961 | 3180.518 | 3250.368 | 3258.937 |
| lognormal | 3181.091 | 3189.648 | 3265.813 | 3274.382 |
| **loglogistic** | **3161.904** | **3170.461** | **3246.259** | **3254.828** |

**Table S3** AIC and BIC values of PFS curve fitting distributions for the two treatment groups

| **Model** | **Pbgemcis, AIC** | **Pbgemcis, BIC** | **Gemcis, AIC** | **Gemcis, BIC** |
| --- | --- | --- | --- | --- |
| exponential | 2314.9247 | 2319.202 | 2365.437 | 2369.721 |
| Weibull | 2306.136 | 2314.693 | 2343.160 | 2351.728 |
| Gamma | 2301.637 | 2310.194 | 2334.599 | 2343.168 |
| **lognormal** | **2291.845** | **2300.402** | **2316.106** | **2324.674** |
| loglogistic | 2295.032 | 2303.589 | 2325.397 | 2333.965 |

**Table S4** The range of key model inputs (China and Japan)

| **Traeatment cost** | **China** | **Ranges** | **Japan** | **Ranges** |
| --- | --- | --- | --- | --- |
| Pembrolizumab(200mg) | 5034.63 | 4027.70-6041.56 | 2817.71 | 2254.17-3381.25 |
| Gemcitabine (0.2g) | 42.13 | 33.70-50.56 | 6.11 | 4.89-7.33 |
| Cisplatin (10mg) | 12.08 | 9.66-14.49 | 22.09(50mg) | 17.67-26.51 |
| Fluorouracil (250mg) | 25.06 | 20.05-30.07 | 1.58 | 1.26-1.89 |
| Folinic acid (0.1g) | 25.59 | 20.47-30.71 | 2.99 | 2.39-3.59 |
| Oxaliplatin (50mg) | 13.28 | 10.62-15.93 | 18.10 | 14.48-21.72 |
| **Adverse event cost, $** |  |  |  |  |
| Neutropenia | 354.00 | 283.20-424.80 | 244.62 | 195.69-293.54 |
| Leukopenia | 466.00 | 372.80-559.20 | 163.03 | 130.42-195.64 |
| Thrombocytopenia | 1814.00 | 1451.20-2176.80 | 803.63 | 642.90-964.36 |
| Anemia | 541.00 | 432.80-649.20 | 15.00 | 12.00-18.00 |
| **Other cost, $(per cycle)** |  |  |  |  |
| Follow-up | 55.60 | 44.48-66.72 | 115.67 | 92.54-138.80 |
| Administration | 110.20 | 88.16-132.24 | 620.00 | 496.00-744.00 |
| Best Supportive Care | 337.50 | 270.00-405.00 | 79.85 | 63.88-95.82 |
| End-of-Life Care | 2299.00 | 1839.20-2758.80 | 12628.56 | 10102.85-15154.27 |
| **Uyility valus** |  |  |  |  |
| PFS | 0.76 | 0.61-0.91 | 0.73 | 0.58-0.88 |
| PD | 0.68 | 0.54-0.82 | 0.69 | 0.55-0.83 |

**Table S4 (Continued)** The range of key model inputs (US and CH)

| **Traeatment cost,**  **($ per cycle)** | **US** | **Ranges** | **CH** | **Ranges** |
| --- | --- | --- | --- | --- |
| Pembrolizumab(200mg) | 10,684.30 | 8,547.44-12,821.16 | 10,798.56 | 8,638.85-12,958.27 |
| Gemcitabine (0.2g) | 4.00 | 3.20-4.80 | 41.36 | 33.08-49.63 |
| Cisplatin (10mg) | 3.00 | 2.56-3.84 | 35.53 | 28.42-42.64 |
| Fluorouracil (250mg) | 2.10 | 1.68-2.52 | 15.55(500mg) | 12.44-18.66 |
| Folinic acid (0.1g) | 8.31 | 6.65-9.97 | 45.01 | 36.01-54.01 |
| Oxaliplatin (50mg) | 6.00 | 4.80-7.20 | 112.03 | 89.62-134.44 |
| **Adverse event cost, $** |  |  |  |  |
| Neutropenia | 5,321.00 | 4,256.80-6,385.20 | 6,845.62 | 5,476.49-8,214.74 |
| Leukopenia | 5,321.00 | 4,256.80-6,385.20 | 9,307.84 | 7,446.27-11,169.41 |
| Thrombocytopenia | 6,325.00 | 5,060.00-7,590.00 | 6,845.62 | 5,476.49-8,214.74 |
| Anemia | 4,953.00 | 3,962.40-5,943.60 | 6,845.62 | 5,476.49-8,214.74 |
| **Other cost, $** |  |  |  |  |
| Follow-up | 851.00 | 680.80-1021.20 | 659.79 | 527.83-791.75 |
| Administration | 2,046.00 | 1,636.80-2,455.20 | 1,198.83 | 959.06-1,438.59 |
| Best Supportive Care | 222.60 | 178.08-267.12 | 249.82 | 199.86-299.78 |
| End-of-Life Care | 7,894.00 | 6,315.20-9,472.80 | 14,101.41 | 11,281.13-16,921.69 |
| **Uyility valus** |  |  |  |  |
| PFS | 0.76 | 0.61-0.91 | 0.80 | 0.64-0.96 |
| PD | 0.68 | 0.54-0.82 | 0.73 | 0.58-0.88 |
| **Probability of AEs in pem group** |  | | | |
| Neutropenia | 0.47 | | | 0.38-0.56 |
| Leukopenia | 0.12 | | | 0.09-0.14 |
| Thrombocytopenia | 0.16 | | | 0.13-0.19 |
| Anemia | 0.24 | | | 0.19-0.29 |
| Probability of AEs in the chem group |  | | | |
| Neutropenia | 0.46 | | | 0.37-0.55 |
| Leukopenia | 0.09 | | | 0.07-0.11 |
| Thrombocytopenia | 0.18 | | | 0.14-0.22 |
| Anemia | 0.25 | | | 0.20-0.30 |

**Table S5** Scenario analysis

| **Reduction in China (%)** | | **ICER** | **Reduction in Japan (%)** | **ICER** | **Reduction in US (%)** | **ICER** | **Reduction in CH (%)** | **ICER** |
| --- | --- | --- | --- | --- | --- | --- | --- | --- |
| 10 | 492,123.42 | 10 | 285,353.42 | 10 | 998,907.87 | 10 | 968,232.30 |  |
| 20 | 441,805.68 | 20 | 256,493.16 | 20 | 893,187.35 | 20 | 866,967.77 |  |
| 30 | 391,487.94 | 30 | 227,632.91 | 30 | 787,466.82 | 30 | 763,220.83 |  |
| 40 | 341,170.20 | 40 | 198,772.66 | 40 | 681,746.30 | 40 | 664,438.34 |  |
| 50 | 290,852.46 | 50 | 169,912.40 | 50 | 576,025.78 | 50 | 563,174.19 |  |
| 60 | 240,534.72 | 60 | 141,052.15 | 60 | 470,305.25 | 60 | 461,909.66 |  |
| 70 | 190,216.98 | 70 | 112,191.89 | 70 | 364,584.73 | 70 | 360,645.13 |  |
| 80 | 139,899.24 | 80 | 83,331.64 | 80 | 258,864.20 | 80 | 259,380.61 |  |
| 90 | 89,581.50 | 90 | 54,471.38 | 90 | 153,143.68 | 90 | 158,116.08 |  |
| 95 | 64,422.63 | 95 | 40,041.26 | 95 | 100,283.42 | 95 | 107,483.81 |  |

| (a) | (b) |
| --- | --- |
| **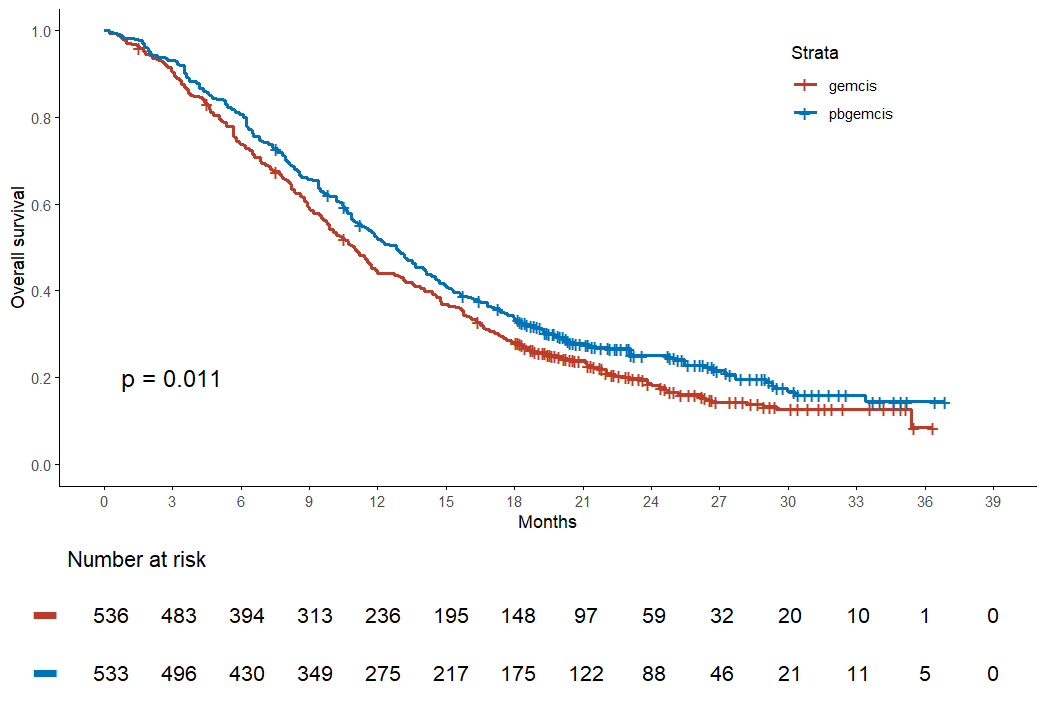** | **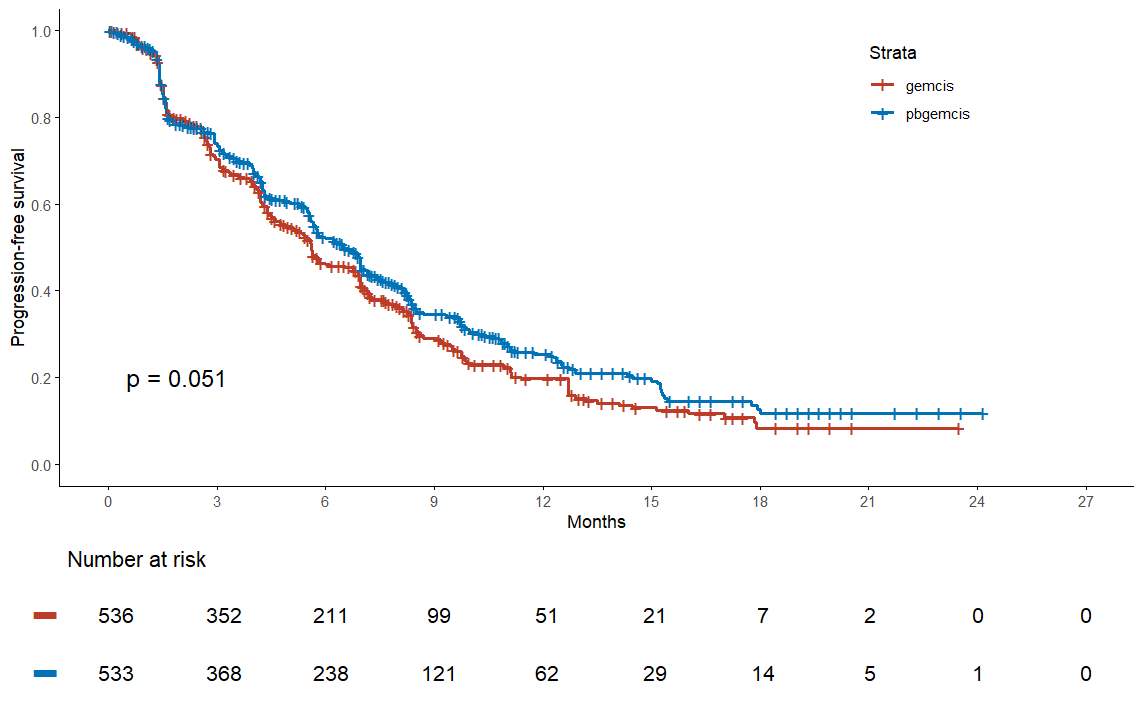** |

**Fig S1.** OS and PFS Curves Reconstructed from the KEYNOTE-966 Phase III Trial. **a** Reconstructed overall survival (OS) curve. **b** Reconstructed progression-free survival (PFS) curve

| (a)   |
| --- |
| (b) |
|  |
| (c) |
| **** |

**Fig S2.** Extrapolation Curves of Different Statistical Distributions. **a** Extrapolated PFS Curves for Pembrolizumab + Gemcitabine + Cisplatin. **b** Extrapolated OS Curves for Gemcitabine + Cisplatin. **c** Extrapolated PFS Curves for Gemcitabine + Cisplatin

| 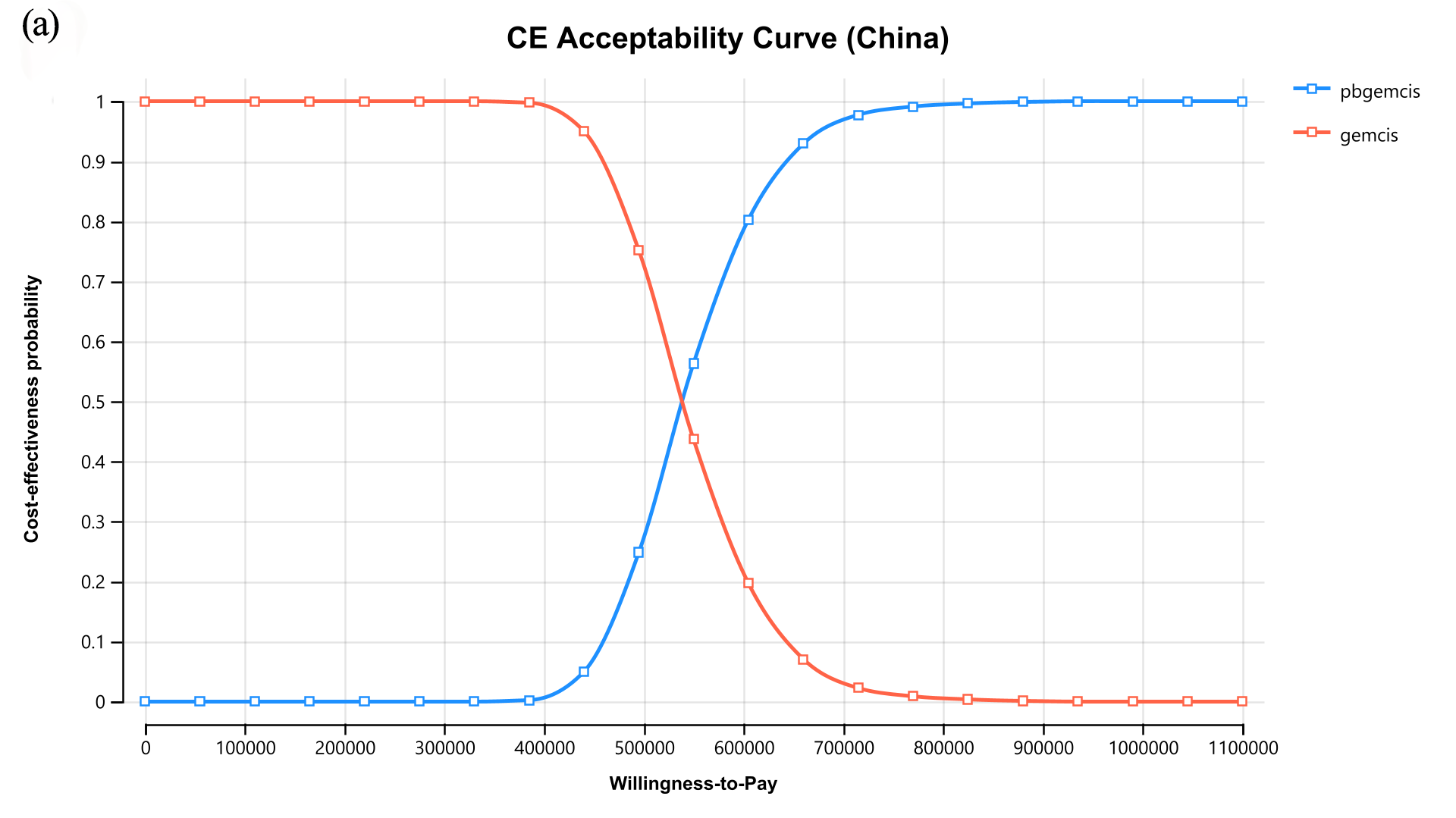 | 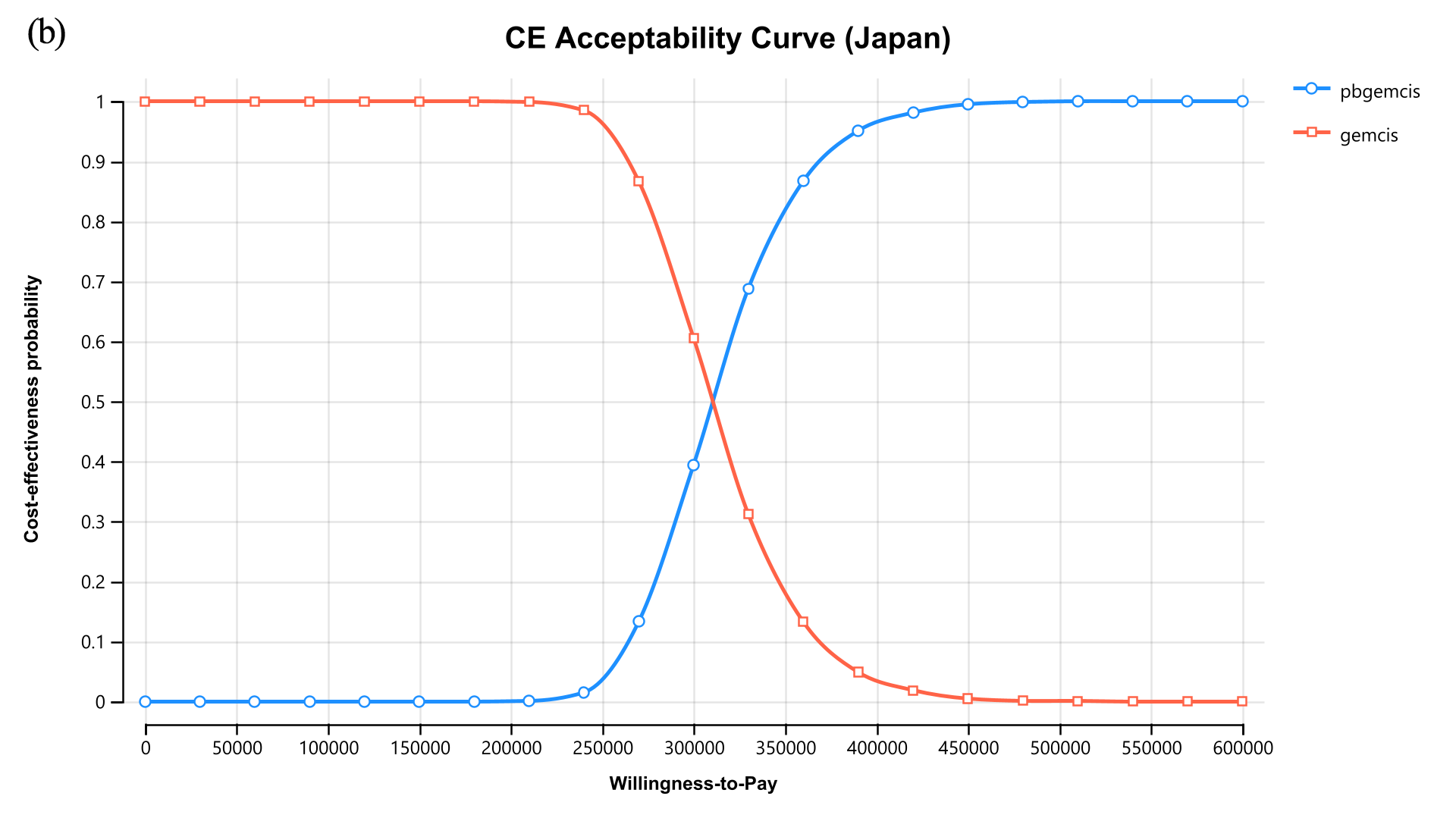 |
| --- | --- |
| 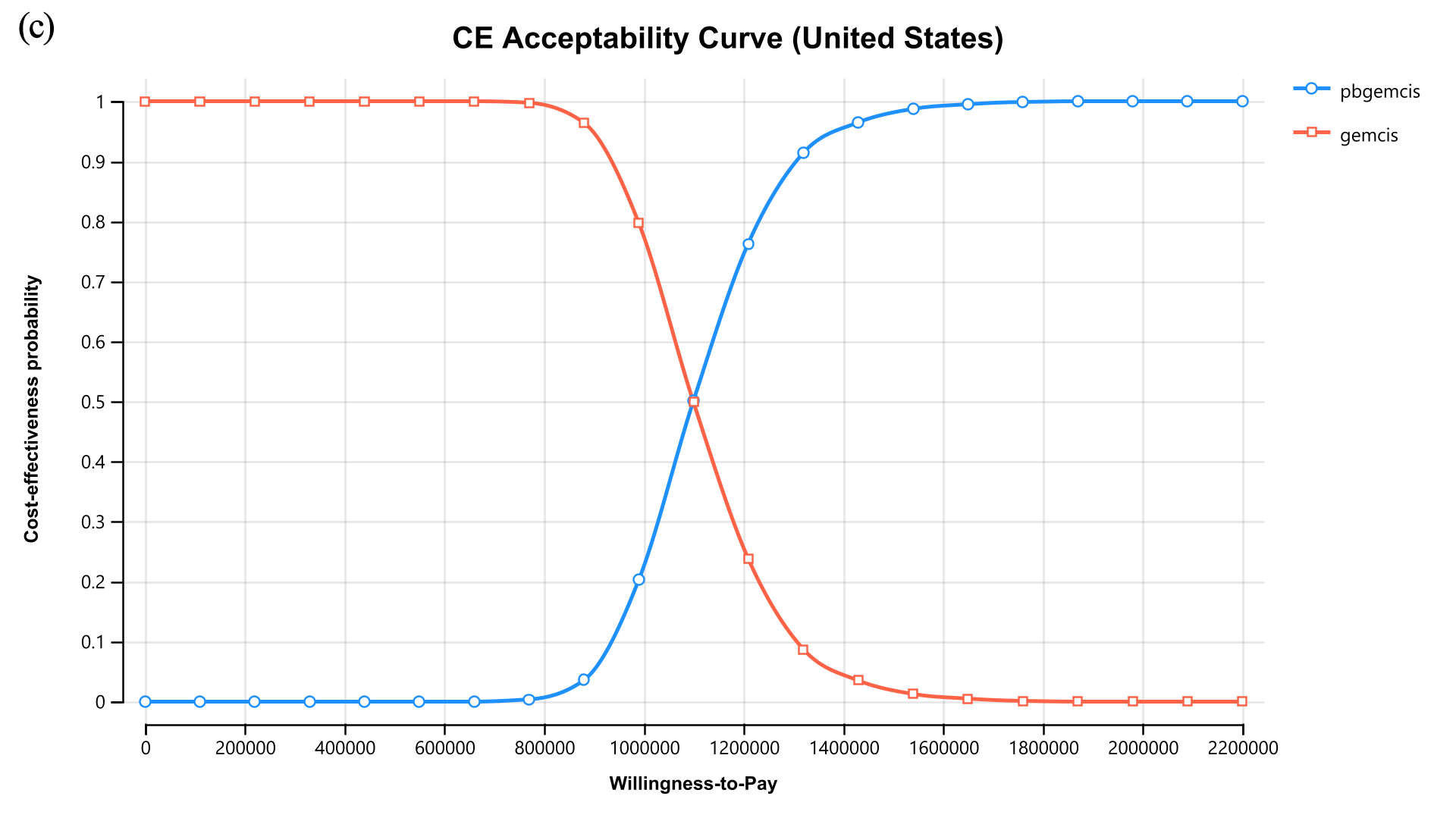 | 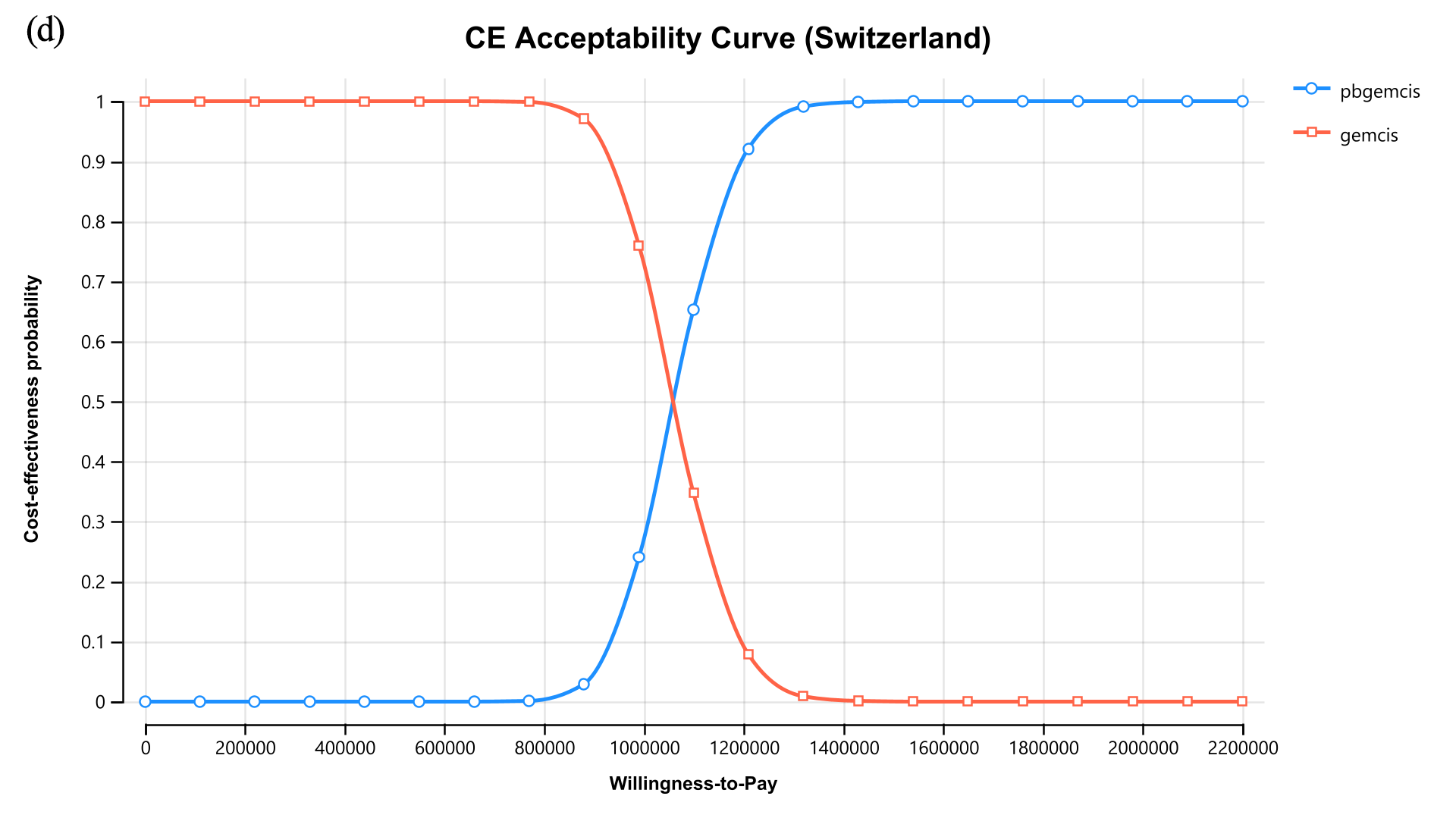 |

**Fig S3.** Probability sensitivity analysis (CEACs)
